# Adapting emerging digital communication technologies for resilience: evidence from Nigerian SMEs

**DOI:** 10.1007/s10479-022-05049-9

**Published:** 2022-11-23

**Authors:** Folajimi Ashiru, Franklin Nakpodia, Jacqueline J You

**Affiliations:** 1 School of Strategy and Leadership, Coventry University, Coventry, UK; 2 Accounting Department, Durham University Business School, Durham University, Durham, UK; 3 Department of Financial Intelligence, University of South Africa, Pretoria, South Africa; 4 School for Business and Society, University of York, York, UK

**Keywords:** Emerging digital communication technologies, Diffusion of innovation, Disruptions, COVID-19, Nigeria, Weak institutional environment

## Abstract

Drawing on the Diffusion of Innovation Theory, this study explores how emerging digital communication technologies (EDCT) affected SMEs’ resilience during the COVID-19 pandemic. We employed an inductive and qualitative approach to investigate 42 SME operators in a weak institutional developing country—Nigeria. Our findings show that EDCT played a critical role in activating SMEs’ resilience during the crisis through four drivers: facilitating connections and bonding with staff, clients, and suppliers; enabling collaborations; activating process diversification; and enhancing supply chain flexibility. Furthermore, we highlight the distinct ability of Nigerian SMEs to buffer themselves against misinformation arising from the use of EDCT. This study sheds light on an EDCT Diffusion Model for resilience.

## Introduction

The COVID-19 crisis triggered supply chain (SC) disruptions^1^ for businesses such that firms found it challenging to continue in business (You, 2021), especially SMEs (Markovic et al., 2021). Under crisis situations, emerging digital technologies^2^ are relevant in dealing with SC disruptions (Kim & Dennis, 2019; Olan et al., 2022). This is because emerging digital technologies are critical in managing information dissemination and allow agents to make decisions when tackling complex issues (Endsley, 2018; Jayawickrama et al., 2019). Given the fundamental role of emerging digital technologies in communication and information sharing, limited research has investigated its effects on SC disruptions in the areas of misinformation (Kim & Dennis, 2019), disruption of values, and meanings (Rauch & Ansari, 2021) and, more broadly, for resilience during crises such as the COVID-19 pandemic (Olan et al., 2022). This scholarly neglect is particularly worth noting for SMEs, as their size and resource limitations impact their activities, inhibit their ability to adopt emerging digital technologies, and challenge their capacity to obtain reliable information during crises (Eggers, 2020). These concerns are constraining, especially as the ability to exchange information promptly in SCs is a key driver of resilience (Kamalahmadi & Parast, 2016). Following this logic, this research focuses on how SMEs adapted emerging digital communication technologies (EDCT) to overcome SC disruptions during the COVID-19 crisis. Considering the resource constraint of SMEs, this study focuses on social media and virtual communications technologies such as EDCT media.

Across developed economies, EDCT has become an influential contemporary business buzzword in the context of social systems and policy (Fougère & Meriläinen, 2021). EDCT enhances SMEs’ performance in these settings, offering them greater opportunities to compete with their larger counterparts (Cenamor et al., 2019). However, in many developing countries, the influence of EDCTs in businesses remains rudimentary (Fougère & Meriläinen, 2021). Moreover, as many of the markets in developing countries suffer from weak institutions (Kaufmann et al., 2008),^3^ SMEs from these contexts encounter significant growth and development challenges, notably in their communication and information architecture (Koporcic et al., 2015). These challenges exacerbate during disruptions and crises. EDCT provides vast opportunities for SMEs to overcome underlying institutional challenges (Bagale et al., 2021). Surprisingly, there is less evidence of how EDCT impact SMEs’ operations in developing countries during disruptions such as the COVID-19 pandemic (Ashiru et al., 2022; Bagale et al., 2021). For example, how SMEs in developing countries adapted digital technologies or innovations in their SC during COVID-19 is empirically under-researched (Aman & Seuring, 2021; Ashiru et al., 2022; Chesbrough, 2020). Resilience, defined as the ability to sustain reliable functioning despite crisis or adversity (Williams et al., 2017), accelerates ‘an interactive process of relational adaptation’ (Williams et al., 2017, p.742) with the environment, including understanding and responding to variations, as well as making positive adjustments. While SC disruption and resilience literature abound (e.g., Papadopoulos et al., 2017; Pettit et al., 2019; Soni et al., 2014; Waller & Fawcett, 2013), the scholarship focuses on a high-level view of resilience among large firms. The literature has paid minimal attention to how SMEs manage disruption and whether utilising EDCT can enhance their resilience to disruption and crisis (Kamalahmadi & Parast, 2016), particularly in weak institutional contexts (Aman & Seuring, 2021; Luo et al., 2021).

Disruption stimulates the process of innovation for both technology and markets, and EDCT can offer a medium of value utility in markets (Vargo et al., 2015). In this regard, the diffusion of innovation theory (DOI) explores how change develops in environments through the adaptation and innovation of social agents. Hence, the theory can explain resilience developed through value utility (Atwell et al., 2008). The theory further suggests that the beliefs and attitudes of agents are crucial to the diffusion of innovative products such as EDCT. However, supporting empirical studies are sparse (Franceschinis et al., 2017) and even less so in weak institutional settings (Fougère & Meriläinen, 2021). Considering the need to understand EDCT’s role in facilitating SMEs’ operations during the COVID-19 crisis, we utilise the DOI to address the question: *How did EDCTs enable SMEs in a weak institutional context to adapt their operations and build resilience during the COVID-19 pandemic-induced SC disruptions?*

We address this question by conducting in-depth semi-structured interviews with forty-two (42) SME operators in Nigeria. SMEs play a critical role in supporting global supply chains (GSCs), and their inability to function could damage the entire GSC (Rice & Caniato, 2003). Our context is appropriate because scholars such as Khanna et al., (2005) posit that developing economies (e.g., Nigeria—the 27th largest economy in the world with a GDP of USD448BN, according to the World Bank, 2020) have a high potential due to their market size and their attractiveness as investment destinations. SMEs, which are critical to Nigeria’s economy, faced considerable SC disruptions during the COVID-19 pandemic and had to rely on their relational capabilities (Ashiru et al., 2022). This is because the Nigerian government, like many other governments, imposed a lockdown of activities to curtail the spread of the pandemic. Yet, unlike some other countries, institutional support in Nigeria during the crisis was limited (Ashiru et al., 2022). Therefore, in these highly competitive but less-supported business environments, firms need to generate relevant innovations and technologies to boost their performance and competitiveness (Iglesias et al., 2020; Markovic et al., 2021). It is worth noting that the Nigerian government became one of the first governments worldwide to adopt a digital electronic currency (Daily Trust, 2021). This shows that despite the rudimentary nature of Nigeria’s business environment, individuals and businesses are familiar with digital technologies, especially communication technologies that were initially intended to facilitate connections between friends but are now deployed for producing and exchanging news and information (Tandoc et al., 2018).

This study contributes to the literature in three ways. First, we apply the DOI by demonstrating how social agents can adapt emerging digital technologies for beneficial purposes. Specifically, we propose an EDCT Diffusion model for SMEs’ resilience, an innovation that emerged in Nigeria due to the familiarity with existing EDCTs and the lack of viable alternatives. In doing this, our study aligns with the literature (e.g., Dearing & Cox, 2018; Vargo et al., 2015, 2020; David West et al., 2021), which theorises contextually about the utility of diffusion of innovations in an empirical way and with practical implications. Second, we contribute to the empirical literature on emerging digital technologies and resilience (e.g., Bagale et al., 2021; Olan et al., 2022) by developing knowledge and generating insights into the significance of EDCT for SMEs during the COVID-19-induced SC disruptions. Our findings show that EDCT enabled SMEs to be resilient amidst SC disruptions via four drivers—facilitating connections and bonding with staff, clients, and suppliers; enabling collaborations; allowing process diversification; and, permitting SC flexibility. Finally, we contribute to literature (e.g., Fougère & Meriläinen, 2021; Markovic et al., 2021) investigating the effects of innovation on information during disruptions and crises in developing economies. It is noteworthy that this study finds that during the COVID-19 crisis, SMEs in Nigeria demonstrated a high level of resistance to the adverse effects of EDCTs (e.g., facilitation of fake news and misinformation). This is because Nigerian SMEs are constantly exposed to unreliable information aided by weak institutions (Kaufmann et al., 2008). This paper details how existential fears, prompted by the COVID-19 pandemic, made SMEs relegate the negative features of EDCT to the background.

The rest of this study is organised as follows. Section 2 reviews the literature underpinning this research and introduces the theoretical framework. We present the research methodology in Sect. 3, while findings and discussions are detailed in Sects. 4 and 5. Section 6 concludes this paper together with its limitations and future research suggestions.

## Literature review and theory

### Supply chain disruptions, the COVID-19 pandemic and resilience

Before the COVID-19 pandemic, the globalisation of procurement and distribution activities had disrupted GSCs (Pettit et al., 2019), making SCs more complex and vulnerable (Hendricks & Singhal, 2005). During the COVID-19 pandemic, governments’ preference to pay attention to health emergencies at the expense of other economic considerations compounded SC vulnerability (Aman & Seuring, 2021). In contemporary times, quick reaction and satisfying customer needs are considered SC threshold capabilities (Modrak et al., 2019; Spath et al., 2013). As such, the COVID-19 pandemic-induced disruptions to SCs are noteworthy (Aman & Seuring, 2021). Researchers (e.g., Hohenstein et al., 2015; Tukamuhabwa et al., 2017) argue that disruptions to SCs can be managed by building resilience to ensure continuous delivery of goods and services to customers (Scholten & Schilder, 2015). During crises such as COVID-19, SC resilience helps firms survive disruptions and improves their ability to adapt and grow (Gabler et al., 2017; Ivanov et al., 2014). Kamalahmadi and Parast (2016) note that enterprise resilience emphasises firms’ dynamic capability, which relies on its individuals, groups, and systems to deal with immediate and unexpected environmental changes with a proactive attitude and to adapt and respond to these changes by developing flexible and innovative solutions. Leading from this, SC resilience practices highlighted in the literature include information sharing (Brandon-Jones et al., 2014; Kamalahmadi & Parast, 2016), collaborations (Scholten et al^2014^; Soni et al., 2014), and flexibility and reconfiguration of resources (Ambulkar et al., 2015; Scholten & Schilder, 2015), among others. However, the existing literature concentrates on developed countries (Aman & Seuring, 2021).

Unlike developed countries, developing economies lack efficient operational systems due to deep-rooted infrastructural challenges (Aman & Seuring, 2021; Rehman et al., 2020). Consequently, firms in developed countries build robust resilience compared to those in developing countries. For example, the average total insurance premiums as a percentage of GDP in developed countries is twice as high as those of developing economies, with countries like Indonesia, Egypt and Nigeria having insurance penetration rates of less than 1% (Lloyds, 2018). This is concerning given that firms in developing countries with weak institutions are more susceptible to SC risks such as political instability, wars and rebel activities, bribery and corruption, poor transportation infrastructure, corruption and other unethical business practices (Transparency International, 2018). Therefore, in weak institutional settings, the unique crisis requires nuanced contextual resilience concepts that can help overcome known vulnerabilities (Aman & Seuring, 2021). Recently, empirical studies on SC resilience in developing countries have begun to emerge. Using a case study approach, Tukamuhabwa et al., (2017) conducted 45 interviews with 20 manufacturing firms in Uganda. Their study reports that threats of disruption are side-effects of strategies, and building resilience within the SC requires intervention and adaptation. Also, Aman and Seuring (2021) conducted a mixed-methods study in Pakistan, India and Iran, articulating 36 resilience categories. While their findings suggest that SC disruption is a significant vulnerability for developing countries, they note that solutions to disruptions lie in reconfiguring resources, such as social capital, financial, technological, human, information, and materials.

Considering the high labour dependence in developing economies, the rapid development and adoption of technologies profoundly impact how SCs operate. Pananod et al., (2020) point out that the emergence of digital platform-based enterprises (e.g., Amazon, Alibaba) has removed intermediaries between producers and consumers on the demand side and lowered the entry barriers for SMEs to access global markets on the supply side. Resilience developed through EDCT can be interpreted as an SC capability that complements the traditional risk processes, which could offset the severity of SC vulnerabilities in weak institutional contexts (Pettit et al., 2013). EDCT allows for greater customer personalisation, increased versatility, and SC globalisation (Baum, 2013). Though Williams et al., (2017) and Aman and Seuring (2021) identify cognitive, behavioural, emotion regulation, and relational resource endowments as the building blocks of durability capabilities, the literature is yet to explore how EDCTs enhance SMEs’ inter-organisational and business relationships resilience during crisis-induced (e.g., COVID-19) SC disruptions.

### Emerging digital communication technologies and resilience

The COVID-19 pandemic exemplifies how emerging digital technology facilitates communication and collaboration among agents in GSCs, but how these technologies produce practical solutions for firms varies across contexts. Therefore, there is a need to understand the market (Bagale et al., 2021) and contextual attendant risks (DuHadway et al., 2018). Moreover, since SMEs have resource limitations, their capacity to source knowledge or information is restricted compared to large firms, which informs their reliance on readily accessible technologies (Jayawickrama et al., 2019; Masouras et al., 2021). As this research investigates SMEs in a Sub-Saharan African country, we explore social media and virtual communication technologies (e.g., zoom) as EDCTs.

Social media and virtual communication technologies are emerging digital technology platforms originally intended to enable connections between friends but are now critical to producing and exchanging news and information (Tandoc et al., 2018). These communication platforms are easily accessible by SMEs. The use of social media and virtual communication platforms is cost-effective and typically produces greater efficiency than traditional communication methods (Kaplan & Haenlein, 2010; Omotosho, 2020). Indeed, several studies affirm the importance of EDCT to businesses. Analysing data from 453 SME managers, Fosso Wamba and Carter (2017) investigate SMEs’ adoption of social media tools and find that firm innovativeness, firm size, manager’s age, and industry sector significantly impact social media adoption. Similarly, Srinivasan et al., (2016), relying on data from 50 Micro, Small and Medium Enterprises (MSMEs), observe that social media plays a crucial role in establishing brand reputation and generating brand awareness. They further note that social media engenders customer growth, acquisition, and brand recognition. Recent research by Bagale et al., (2021) supports Fosso et al., (2017) and Srinivasan et al., (2016) claims, reinforcing that social media assists in retaining and building strong customer relationships to create innovative markets and enhanced market share. Furthermore, EDCTs help combat disruptions and crises. Using unstructured Big Data involving 36,422 items gathered from tweets, news, Facebook, WordPress, Instagram, Google + , and YouTube, and structured data from 205 managers involved in disaster management following the 2015 Nepal earthquake, Papadopoulos et al., (2017) contend that swift trust, public–private partnerships, and information sharing through social media, support SC resilience during a crisis. Nevertheless, there is a need to understand how firms deal with SC complexities during crises to minimise global commerce and community life disruptions (Day et al., 2012). Therefore, it is critical to understand how EDCT is utilised during crises (Hazen et al., 2014).

While we have highlighted the benefits of EDCTs, they still pose specific risks to SCs. Though the value of EDCT as a crucial channel for information and marketing (Tajvidi et al., 2018) and customer service and product development (Baccarella et al., 2018) is growing, these technologies are criticised for their role in disseminating misinformation or fake news^4^ (Berthon & Pitt, 2018). Researchers (e.g., Di Domenico et al., 2021; Papadopoulos et al., 2017; Pettit et al., 2019) inform that misinformation activates disruptions to business activities, including SC, innovation and even loss of life. Misinformation can tarnish firms’ reputations (Berthon & Pitt, 2018) and threaten their financial stability and resilience (Binham, 2019). In essence, EDCT can emotionally redefine the entire context of business, positively or negatively (Rauch & Ansari, 2021). Following the harsh effects of COVID-19, it is important to understand how the negative aspects of EDCT impact SCs (Kim & Dennis, 2019), especially for SMEs in a weak institutional context.

### SMEs and the COVID-19 pandemic in weak institutional contexts

There is little evidence of how SMEs in developing countries utilised technology to build SC resilience in response to the COVID-19 crisis (excluding Markovic et al., 2021; Sengupta et al., 2021). Still, technology is effective in SC resilience building in developing countries because these countries are immature in controlling the sources of disruptions (Sengupta et al., 2021). Also, SMEs usually have labour-intensive operations, making them more vulnerable to crises (Gunasekaran et al., 2011). In this regard, Borges et al., (2021) assert that emerging technologies enable SMEs to resolve man labour issues, manage operational costs, decrease overall expenses, and cope with the COVID-19 challenge. Polanco-Diges and Debasa (2020) add that utilising emerging technologies spurs productivity, as SMEs can digitalise their processes, transform all paper documents into e-Documents, and migrate onto e-commerce services. This helps mitigate operational challenges and minimise red tape (Polanco-Diges & Debasa, 2020). Thus, due to their meagre resources, EDCTs have become even more crucial for SMEs.

In developing economies such as Nigeria, SMEs are aware of social media platforms and their relevance for business endeavours (Omotosho, 2020). However, there is a lack of platform continuity for business (due to maintenance costs, fraud and misinformation, and poor infrastructure), such that most individuals use social media platforms for pleasure (Omotosho, 2020). Nonetheless, EDCTs offer firms an efficient and effective outlet to deliver and meet their clients’ needs to achieve their profit objectives (Fischer & Reuber, 2011). The literature establishes that social media provides a ready and inexpensive tool that SMEs can deploy to communicate with customers as well as for internal communication and collaboration. Oni (2021) suggests that Web 2.0 technologies and associated social media applications, including social network sites, microblogging, and similar technologies, improve communication and collaboration among employees and customers. Polat and Yarimoglu (2018) interviewed 30 Turkish SMEs to determine their main promotional activities and how they conducted their activities. Their findings reveal that SMEs prefer using social media for their promotional activities due to ease, speed, and convenience. Despite this, there is limited knowledge about the reality of the relationship between EDCTs and business developments during crises, as the literature emphasises the relationship of digital communication platforms with marketing or misinformation.

Furthermore, SMEs in weak institutional environments are relatively less prepared than larger organisations to cope with disruptions (Bak et al., 2020). Despite the importance of SMEs to GSCs, the literature less understands how SMEs navigate GSC disruptions, considering that these disruptions unfold in various forms (e.g., poor communication, transportation delays, quality issues, supply shortage, IT failures) (Blackhurst et al., 2005; Williams & You, 2018). Moreover, in weak institutional countries, coping strategies available to SMEs are limited and sometimes characterised by informality, inadequate management tools and noncompliance with known industry norms and regulations (Bak et al., 2020). Thus, during crises such as COVID-19, SMEs’ coping mechanisms depend on the SC’s ability to respond, react, and adapt to contextual and environmental changes (Bak et al., 2020).

In the meantime, the scale of engagement with emerging technologies has become a core metric for assessing organisational knowledge (Oyemomi et al., 2016). Information obtained from emerging digital technologies enables data sharing among SMEs, supporting the efficiency of their operations (Baum, 2013). Emerging digital technologies impact SMEs substantially as it offers opportunities to participate in the global economy while developing their resilience (Borges et al., 2021). For instance, EDCTs permit SMEs to customise activities (e.g., digital marketing) that allow them access to global markets (Bagale et al., 2021). However, the extent to which SMEs deploy emerging technologies across varieties of capitalism remains inconsistent. In developing economies, a considerable lacuna exists in how SMEs integrate emerging technologies (Bagale et al., 2021). In these highly competitive but less supported settings, firms need to generate relevant innovations or technologies to boost their performance and competitiveness (Iglesias et al., 2020; Markovic et al., 2021). During crises, innovations (such as EDCT) can lead to resilience, becoming a ‘new normal’ (Fougère & Meriläinen, 2021). Indeed, against the backdrop of COVID-19, Sengupta et al., (2021) case study shows how an SME in India used emerging technologies to redesign its SC to improve its resilience. Surprisingly, several contextual aspects of emerging technology innovation diffusion typically go unstudied, but adopters of EDCT might have a choice in how they utilise innovations (Dearing & Cox, 2018). It is also instructive that the literature is yet to reflect on how SMEs overcome the negatives of EDCT. Oni (2021) posits that the ability of SMEs to diffuse emerging technology innovations rapidly, to enhance their business value, is debatable. In this regard, diffusion of innovation principles can be used to explain SMEs’ adaptation of EDCT for beneficial purposes (Dearing & Cox, 2018).

### Theoretical underpinning (diffusion of innovation)

ROGERS’ (2003) seminal framework on the diffusion of innovation underpins diffusion as a communication process. In this innovation-decision-making diffusion process, Rogers (2003) articulates five steps: (1) knowledge, (2) persuasion, (3) decision, (4) implementation, and (5) confirmation**.** In the knowledge stage, actors learn about the existence of innovation and seek information about the innovation. In the persuasion stage, actors form a favourable or unfavourable attitude towards the innovation (Rogers, 2003). In this persuasion stage, five characteristics of innovations could be used as indicators to assess usefulness perceptions. These are *relative advantages* (the degree to which an idea is considered better than existing ideas and is more economically profitable), *compatibility* (the extent to which past innovation is considered consistent with existing values, past experiences, and adopters’ needs), *complexity* (the extent to which an innovation is considered relatively difficult to understand and use), *trialability* (the degree to which an innovation can be experimented with, on a small scale) and *observability* (the extent to which adopters can easily identify innovations) (David-West et al., 2021; Rogers, 1983). At the decision stage, actors choose to adopt or reject the innovation. The innovation-decision diffusion process also consists of the implementation stage—where innovation brings the newness in which “some degree of uncertainty is involved in diffusion” (Rogers., 2003, p. 6) and the confirmation stage, where actors look for support for their adoption decision.

In addition, the DOI posits that the adopters’ perceptions of innovation attributes influence their adoption decisions. Per DOI, diffusion of innovation is a social phenomenon with four aspects—the demand to adopt the innovation; communication through specific channels; communication among individuals in a social network; and communication over time (Rogers, 1995; Talebian & Mishra, 2018). For EDCTs, widespread use and acceptance in a social context are critical in influencing users’ adoption decisions (Prescott & Conger, 1995). Importantly, it is the adopters’ perceptions of the utility of the innovation that influence adoption rather than some expert’s assessment of the factors (Rogers, 1995; Van Slyke, Belanger & Communale, 2004). Similarly, in adapting emerging digital technologies for different purposes (e.g., resilience), the perception of the utility of the technology drives its adaptation (Van Slyke et al., 2002). In this instance, few studies have integrated resilience and DOI. Using 33 in-depth interviews, Atwell et al., (2008) integrate resilience and diffusion of innovation frameworks to deepen our understanding of how stakeholders make decisions amid an uncertain future. Their research shows that the adoption of innovative practices is based not only on immediate profitability but also on the interplay between multiscale contextual drivers. Consequently, though emerging digital technologies substantially impact SMEs and their ecosystems as they offer new opportunities and stimulate participation in the GSC (Bagale et al., 2021; Olan et al., 2022), their adoption, adaptation and usefulness depend on diverse drivers in different contexts.

Leading from the above, the nature of emerging digital technologies and social context defines the adoption of innovation (Rogers, 1983). Nevertheless, innovations do not necessarily diffuse quickly in developing countries, and less so among SMEs (Oni, 2021). Mochoge (2014) conducted an explanatory study, relying on data from 396 respondents covering 98 Kenyan SMEs. Employing theories of technology adoption, planned behaviour and diffusion of innovation, Mochoge (2014) demonstrates that understanding plays a significant role in the SMEs’ adoption of new technologies. The study adds that the perceived ease of use, perceived cost, and perceived utility positively influence how web-based marketing resources are adopted. In another study that identifies distinct digital marketing capabilities in industrial firms, Herhausena et al., (2020) adopted the resource-based perspective as an organising framework and reviewed 129 articles spanning two decades. They stress-tested knowledge of emerging technologies from four themes (channels, social media, digital relationships, and digital technologies), identified by conducting an online survey among 169 SME managers. Their investigation suggests a discrepancy between managers’ ‘current’ practices and their ‘ideal’ digital marketing capabilities. They note that the knowledge gap reveals a sizable divide between digital marketing transformations in firms and the underlying scholarly knowledge. Also, Pradhan et al., (2018), utilising DOI, explored all the research points related to Indian SME studies of digital marketing published between 2005 and 2016. Though they find a continuous development of digital advertising, they note that minimal progress occurred among Indian SMEs.

Overall, it is apparent that, in developing countries, most individuals use social media for hedonic purposes, including seeking entertainment or connecting with friends (Johnson & Kaye, 2015), rather than for work tasks (Kim & Dennis, 2019). However, physical connection with people and work during the pandemic became difficult due to the lockdown. Consequently, the usefulness and diffusion of innovations such as EDCT are dynamic, inclusive, integrative, and dependent on contextual institutions (Vargo et al., 2020). In this sense, the DOI is particularly useful in this research as it does not limit innovation to new technologies but incorporates new ideas and practices (Talebian & Mishra, 2018). In other words, diffusion of innovation requires the consideration of the consequences of innovation on the broader social structure (Vargo et al., 2020). SC resilience developed through EDCT can enable SMEs to manage different types of crises (Rajesh, 2021). Even so, there is a knowledge gap regarding how the utility of EDCT is adapted contextually for resilience during SC crises (Vargo et al., 2020). These EDCTs have allowed a change to a consumer-driven business environment that SMEs have leveraged (Berthon et al., 2007). Besides the enthusiasm surrounding the diffusion of digital technologies, business activities in developing countries have benefitted from these technologies (Oni, 2021). Yet, ample opportunities remain to investigate how SMEs adapt or adopt technologies to build resilience during crises (Kamalahmadi & Parast, 2016). As such, our central research question – *How did EDCT enable SMEs in a weak institutional context to adapt their operations and build resilience during the COVID-19 pandemic-induced SC disruptions?*—is timely.

## Research methodology

Consistent with prior operations management literature (e.g., Papadopoulos et al., 2017; Rauch & Ansari, 2021), a qualitative interpretivist methodology offers a valuable method for exploring the role of emerging technologies during crisis periods. This approach allows us to understand Nigerian SME operators’ experiences before and during the COVID-19 pandemic. The SMEs involved in this study are those that actively engage in SC activities.

### Case context

SMEs in Nigeria present a practical research context for investigating SC management issues during the COVID-19 pandemic. Given Nigeria’s status as a developing economy, these SMEs are critical to its economy. According to the National Bureau of Statistics (NBS, 2021), SMEs in Nigeria contribute 48% of the national GDP. They also account for 96% of businesses and 84% of employment (NBS, 2021). The descriptions of SMEs differ across varieties of capitalism,^5^ elected SMEs had been operating for betw but the NBS clarifies that SMEs in Nigeria should have less than 200 employees. Consistent with this position, this research assumes that SMEs employ less than 200 people. Moreover, the NBS distinguishes SMEs from microenterprises. As of December 2017, the number of microenterprises and SMEs stood at 41,543,028. Micro enterprises were 41,469,947 (99.8%), while those strictly categorised as SMEs were 73,081 (0.2 percent) (NBS, 2021) (see Table 1). Thus, for reliability and considering the low literacy levels and informal business practices in Nigeria, this study focuses on SMEs whose operators have university degrees and comply^6^ with Nigeria’s Corporate Affairs Commission (CAC) business registration laws.

**Table 1:** Statistics of Nigerian SMEs

Total number of SMEs + Microenterprises	41,543,028
Contribution to Nigerian GDP	48%
Percentage of Nigerian businesses	96%
Percentage of Nigerian employment	84%
Number of businesses classified as SMEs	73,081 (0.2% of SMEs + Microenterprises)

### Data collection

This research employs semi-structured interviews to collect data and gain insights that helped address the research objectives. As this approach encourages two-way communication, it offers greater latitude to ask further questions in reaction to what is considered a significant response. Data generated from the interviews provide a deeper understanding and rationale for those beliefs and thoughts rather than enhancing statistical validity (Flick, 2014). The interviews were conducted in the first quarter of 2021. Interviewees were drawn from SMEs registered with the CAC. These SMEs are also members of associations such as Chambers of Commerce, Manufacturers Association of Nigeria (MAN), Women in Business (WIMBIZ), and Advertising Practitioners Association of Nigeria (APCON), among others. Participants were selected from various industrial sectors and four (4) of Nigeria’s six (6) geographical regions. Interviewees with the requisite profile were contacted via emails, WhatsApp messages, and telephone calls. In total, forty-two (42) SME operators were interviewed from twenty-two (22) subsectors (18 service sectors and four manufacturing sectors).^7^ The selected SMEs had been operating for between two (2) to fifty-one (51) years. Their average turnover ranged from N7M to N2.5B ($0.016 M to $5.75 M @ $1 = N435 as of October 16, 2022). Thirty-three (33) interviewees were from service firms, while the remaining nine (9) were from manufacturing companies. As stated earlier, the SMEs are based in four geographical regions (South–West – 34; South–South—4; North-Central—3; North–West—1). Employees range from 1 to 97 full/part-time staff^8^ (see Table 2).

**Table 2:** Respondents and SME Details

Code	Position in company	Sector	Average turnover	Average number of staff (Contract + Full Time)	Years of ope ration	Participant gender	Location
Respondent 1	Owner	Clearing & Forwarding	$2.5 M	31	13	M	Rivers
Respondent 2	Partner/Co founder	IT	$0.25 M	7	4	M	Lagos
Respondent 3	Director	Advertising	$0.97 M	30	11	F	Lagos
Respondent 4	Owner	Travel Agency	$0.20 M	3	3	F	Lagos
Respondent 5	Director	FinTech	$31 M	60	5	M	Lagos
Respondent 6	Director/Daughter of Owner	Furniture Manufacturing	$0.97 M	60	40	F	Ogun
Respondent 7	Co-Owner	Furniture Manufacturing	$0.30 M	6	4	M	Lagos
Respondent 8	Director/Son of Owner	Hotel	$0.61 M	39	51	M	Kano
Respondent 9	Owner	FinTech	$0.50 M	15	3	M	Delta
Respondent 10	Owner	Digital Sales	$0.12 M	10	2	M	Delta
Respondent 11	Senior Partner/Founder	Legal Services	$0.10 M	10	8	M	Lagos
Respondent 12	Owner	Beverage Manufacturing	$0.20 M	40	10	F	Lagos
Respondent 13	Managing Partner/Grand daughter of Founder	School	$0.13 M	75	36	F	Lagos
Respondent 14	Owner	PR Services	$0.25 M	18	9	M	Lagos
Respondent 15	Owner	IT Training	$0.95 M	97	16	M	Lagos/Oyo
Respondent 16	Franchise Owner	IT Franchising	$0.32 M	19	9	M	Various States
Respondent 17	Partner/Co founder	Health Consultants	$0.045 M	10	11	M	Lagos
Respondent 18	Owner	Clothing Sales	$0.37 M	30	14	N	Abuja
Respondent 19	Owner	IT Services	$0.73 M	12	2	M	Abuja
Respondent 20	Manager	Pre-School and Nursery	$0.05 M	15	25	F	Lagos
Respondent 21	Senior Partner/Founder	Oil & Gas Servicing	$6.1 M	92	8	M	Lagos/Port Harcourt
Respondent 22	Owner	Beauty Products Manufacturing	$0.005 M	2	2	F	Lagos
Respondent 23	Owner	Advertising	$0.030 M	3	10	F	Lagos
Respondent 24	Owner	Edutech	$0.005 M	2	2	M	Lagos
Respondent 25	Co Founder	HR Consulting and Training	$0.5 M	10	6	F	Lagos
Respondent 26	Owner	Document Shredding Services and Recyling	$0.048 M	5	4	F	Lagos
Respondent 27	Partner/Co founder	Real Estate	$0.17 M	18	12	M	Lagos
Respondent 28	Managing Partner/Son of Founder	Outdoor Advertising	$1.1 M	11	18	M	Lagos
Respondent 29	Owner	Food Processing and Manufacturing	$0.28 M	2*	2	M	Oyo
Respondent 30	Owner	Food Processing and Manufacturing	$0.50 M	6	5	F	Abuja
Respondent 31	Managing Partner	IT Consultant	$0.25 M	10	2	M	Lagos
Respondent 32	Owner	Swimming Services and Sales	$0.05 M	7	4	F	Lagos
Respondent 33	Managing Director	SAP Consultant/Provider	$0.8 M	12	8	M	Lagos
Respondent 34	Owner	SAP Consultant	$0.25 M	1*	12	M	Lagos
Respondent 35	Managing Partner	Food Manufacturing	$0.11 M	10	6	M	Ogun
Respondent 36	Co-Founder/Managing Partner	Edutech	$2 M	22	11	M	Lagos
Respondent 37	Owner	Consulting	$0.25 M	1*	8	M	Lagos
Respondent 38	Owner	Food Manufacturing	$0.017 M	2	5	F	Ogun
Respondent 39	Owner	Lagal Services	$0.015 M	4	7	F	Lagos
Respondent 40	Co-Owner	Home Moving/Interior Decoration	$0.015 M	2	2	M	Lagos
Respondent 41	Owner	Pharceuticals marketing/production	$4 M	100	10	M	Lagos
Respondent 42	Owner	IT	$0.025 M	3	6	N	Lagos

^*^Use contract/ad hoc staff when needed on projects

As most SMEs are owner-managed, we chose the owner or managing partner as our respondent, as they were in a better position to provide the relevant data for this study. This approach is consistent with the standard recommendation to use the most knowledgeable respondent, especially in SME research (Huber & Power, 1985). The rich data served as a control mechanism for assessing and comparing different views (Adegbite, 2015). Thirty-seven (37) interviews were conducted via Zoom^9^ until saturation^10^ was achieved, but we conducted five (5) additional Zoom interviews to confirm data consistency. There was a very high degree of consistency across respondents’ comments. All interviews were recorded, and each averaged 60–70 min. An interview guide (see Appendix 1) detailing the focus of the interview was used to conduct the interviews. The interview questions were developed following existing literature, especially those based on SC disruptions and digital technologies (e.g., Bagale et al., 2021; Pettit et al., 2019). Our interview methodology is consistent with previous studies on innovation and crises (e.g., Markovic et al., 2021).

### Data analysis

In the first instance, the recorded interviews were transcribed using the Otter.ai transcription software (www.otter.ai). The Otter.ai software is an artificial intelligence and machine learning application that performs speech-to-text transcription and translation. Thus, the software offers simultaneous transcription while interviewing through Zoom (Ashiru et al., 2022; Lobe et al., 2020). Next, the transcribed information was manually reviewed and corrected to aid ‘data immersion’ – a process involving rereading the transcribed text to understand the data better (Bradley et al., 2007). The transcribed interview data generated 546 pages of text. We protected participants’ anonymity to minimise social and business pressures. Data were analysed using the NVivo software package, which allows for the subjective interpretation of the content of text data through a systematic classification process of coding and identifying themes and patterns (Hsieh & Shannon, 2005). Also, NVivo software enables researchers to store all data and codes in a repository that permits a more effective and efficient qualitative data analysis.

**Fig. 1:**
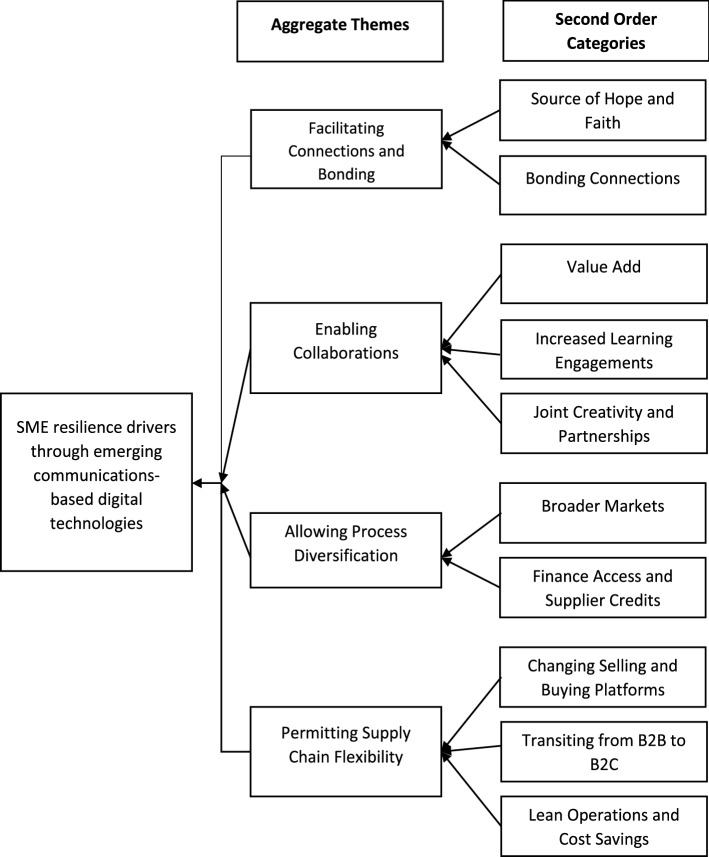
Thematic classifications of findings

Data analysis involved three stages. The first stage focused on generating sub-categories to make sense of the data, followed by an open coding procedure. The open coding stage ensured that the transcribed material was classed into much smaller content categories (Weber, 1990). This process generated themes that represent the sub-categories reflecting participants’ comments. The second data analysis stage involved articulating generic categories where the sub-categories were grouped as higher-order headings (Burnard, 1991). The aim was to reduce sub-categories by combining similar or dissimilar ones into broader higher-order categories (Dey, 2003). This second stage relied on the literature and the theoretical underpinnings of diffusion of innovation. At this stage, we analysed the emerging patterns in our data until adequate conceptual desegregated categories emerged (Eisenhardt, 1989). In the final stage, we followed an abstraction procedure to generate an overall description of the research problem (Nakpodia et al., 2020; Polit & Beck, 2012) that formed the basis of our theorising. Figure 1 shows the second-order categories and aggregate themes, consistent with Gioia et al., (2012).

For triangulation purposes, we confirmed that our final categories were consistent across all 22 SME subsectors and the country’s geographical regions. We refined inductively-derived insights with theoretical insights from the existing literature. Also, to improve data trustworthiness, researchers independently reviewed the data codes and the codes assigned to categories (Campbell et al., 2013). Researchers discussed codes, meanings, and categorisation until an acceptable level of consistency and understanding was achieved. Wherever there was disagreement, categories were modified to maximise inter-coder reliability (Gioia et al., 2010). Finally, to undertake a post hoc analysis, we contacted ten (10) research participants for feedback on our outcomes. These were considerably consistent with our findings.

## Findings

The data analysis identifies how EDCTs are diffused in ways that fuel SMEs’ resilience in a developing country characterised as’a weak institutional environment’ (See Fig. 1). We present, in Sect. 4.1, four themes that emerged from the diffusion and adaptation of EDCTs as resilience drivers—facilitating connections and bonding with staff, clients and suppliers; enabling collaborations; aiding process diversification; and allowing for SC flexibility. In Sect. 4.2, we show why SMEs regard EDCTs as positive outcomes for resilience.

### SME resilience drivers through EDCTs

#### Facilitation of bonding among staff, clients, and suppliers

All respondents confirmed using social media for personal use prior to COVID-19, but mainly for hedonic purposes. However, following the pandemic, the main objectives of EDCTs were to provide hope, a source of faith, and an avenue to bond with stakeholders. Thus, emerging technologies diffused and enabled SMEs to build resilience through (1) a source of hope and faith, and (2) bonding connections.

##### Source of hope and faith

For interviewees, the COVID lockdown meant palpable existential fears. In developing countries with weak institutions, all hopes seem lost for SMEs as there is little or no institutional support. Social media provided an outlet for hope. According to R3 and R5:

*We are a deeply religious people in this country, or at least the places of worship are important to keep sane. Social media interactions became the order of the day, and church services were organised using facetime, Zoom, etc.* (R3).

*Social media and virtual communication gave us hope. It made us realise we are not alone in this crisis, and there is hope for a better future. Hope and faith are critical ingredients for any SME, especially in Nigeria, where governments do nothing for anyone* (R5).

Thus, during COVID-19, EDCTs were adapted as tools for hope and faith.

##### Bonding connection

The majority of respondents bonded more with their stakeholders during COVID-19. R2 states as follows:

*Indeed, our engagement increased despite COVID-19. The communication platforms we adopted meant we kept our people engaged. Whether we had weekly check-in calls, Zoom calls with each other, or to check each other’s welfare.*

For SMEs, EDCTs became “*an in thing*” (R7). R31 explains further:

*We were able to maintain that personal touch virtually during COVID. Zoom and MS Teams parties became a thing. For example, one of our staff was due to marry in April, but the COVID lockdown happened in March. I remember we did a zoom call on her wedding day and stayed with her during the wedding ceremony to keep her spirits up*”.

#### Enabling collaborations

Respondents emphasise the importance of collaborations in building resilience and how EDCTs helped achieve this during the COVID-19 crisis. We coded interviewees’ comments into three areas: value add; increased learning engagements; and, joint creativity and partnerships.

##### Value add

For most respondents, EDCTs enabled them to add value to their businesses. R24 and R17 state as follows:

*Let me give you an idea of the process innovation that virtual communications enabled. During COVID-19, we added value to our clients by teaching them how to utilise Zoom functionalities. We also upgraded their Microsoft knowledge. This is a free value add for clients, and they appreciate it* (R24).

*We made our clients aware of how they can function using social media not only for fun. During our free time, we trained women in small businesses on the use of digital platforms. We did this to remain relevant during and after the COVID-19 crisis* (R17).

For interviewees, adapting EDCTs for value-adding activities during the pandemic assisted them in improving their personal and business resilience.

##### Joint creativity and partnerships

Research participants suggested that EDCTs enabled innovative collaborations, partnerships, and engagements with their business clients. R15 explains thus:

*We were not in the same location, but we were learning from our partners abroad. This made us appear wiser and more in tune with our local clients. We were able to wow our local clients with the business creativity learned virtually through Zoom and GoogleMeet from our overseas clients.*

Similarly, SMEs built resilience by adapting EDCTs in creative ways. As R2 notes:

*Surviving, for us, meant innovations. For example, we gave rebates to our clients. But instead of these rebates being refunded to our clients, we agreed to organise virtual conferences for local charities. Hence, our normal billing time was used in the form of charitable activities*.

##### Increased learning engagements

Finally, respondents identify increased learning as a factor in collaborating for resilience. R39 and R1 state thus:

*Learning was essential during COVID-19. We used social media to cascade important information to clients. I promise you they enjoyed it a lot. Remember, everyone was affected by COVID-19, so constant engagement with clients was our way of collaboration and innovation* (R39).

*Knowledge sharing was key during this (COVID-19) period. Any free time we had was used to update our knowledge. Interestingly, our network widened through social media and virtual presentations* (R1).

The COVID-19-induced lockdowns created free time for firms. Many interviewees utilised this free time to learn and update their knowledge.

#### Allowing process diversification

Before COVID-19, respondents asserted that the business environment in Nigeria required *face-to-face meetings as not all business dealings are official* (R34). However, the global lockdown necessitated a situation where supplies had to be sourced differently and resources utilised in a different way. EDCTs facilitated SMEs’ resilience through process diversification by opening markets across states and cross-borders while enabling access to finance and supplier credit.

##### Opened markets across states and cross-borders

Business dealings and negotiations usually occurred physically before COVID-19. However, the lockdown and SC disruptions meant SMEs had to source materials and market their products through EDCTs. According to R30:

*Although people prefer in-person presentations to virtual ones, we got deals from unexpected places through virtual presentations and remote transactions* (R30).

R21 corroborates thus:

*We expanded the sources of our raw materials outside the state and even outside the country. Now we realise it is possible to get the same things done, if not better.*”

This evidence indicates that EDCTs deepen diversification resilience.

##### Access to finance and supplier credits

Respondents suggest that a lack of access to finance and supplier credits escalated disruption in the SC. Under these SC disruption conditions, emerging technologies enabled SMEs to reshape their business models and payment methods. According to R5:

*How were we able to survive COVID pressures? I can point to the internet and virtual applications. So, before COVID, few of our vendors had online facilities, so we had maybe 30 days timeline for payments of raw materials and products. Everything was manual. But by moving online during COVID-19, we could have accounts receivable and payable days from 30 days to 90/120 days* (R5).

R13 confirms that government finance, which seemed unavailable or difficult to access for SMEs, became accessible.

*The virtual platforms allowed us to access government finance without having to know anyone. This happened only due to COVID-19, as everything moved online. We did not need connections* (*to know people*) (R13).

#### Allowed for supply chain flexibility

According to interviewees, virtual communication platforms made SCs more flexible, allowing them to respond to short-term changes in demand or supply situations occasioned by COVID-19. These EDCTs facilitated: (1) Changing selling and buying platforms/outlets; (2) Moving from Business to Business (B2B) to Business to Customer (B2C); and, (3) allowing for efficient operations and cost savings to deal with external disruptions.

##### Changing selling and buying platforms

Respondents argue that they had to make structural adjustments to survive. R9 describes it as follows:

*We had to think out of the box. We never thought that time would come when everything would be done virtually. Because in our line of business (healthcare), telemedicine was not a thing in Nigeria. Social media and Zoom presentations have helped to overcome this challenge* (R9).

##### Transiting from B2B to B2C

Participants note that to deal with the variable demand from their regular business partners, they utilised EDCTs to reach out directly to customers, thereby expanding their demand source. R21 and R3 put it thus:

*During COVID-19, some of our regular B2B clients stopped our contracts. What did we do? We changed tactics and reached out to consumers directly. Without social media or online platforms, how would we have been able to reach out to the public* (R21)?

*We used social media for booking deliveries and direct delivery to clients’ homes. During the pandemic, social media opened another line of business for us, different from traditional B2B clients* (R3).

##### Efficient operations and cost savings

Respondents contend that emerging technologies allowed for operational efficiency and brought the realisation that costs could be saved regarding staff costs, unnecessary office space and monitoring. R2 and R37 explain as follows:

*We are now more resilient. We learned that we do not need many people to operate, and we do not need large office space as people can work virtually. I could easily monitor and see what people were doing, thereby saving costs* (R2).

*If I give my staff a task, I can track the time it takes to complete the task via email. It works more like timesheets. I can assess their capability and capacity much better, which was not happening pre-COVID-19. We learnt how to measure human capacity in terms of deliverables. We also learnt that we could work remotely from home with minimal disruptions* (R37).

Respondents showed that EDCTs allowed them to institutionalise practices that strengthened SMEs’ resilience. Our follow-up questions to respondents focused on the negative aspects of EDCTs and their impact during the COVID-19 crisis.

### Existential fears leading to backgrounding of the negative aspects of EDCT during the COVID-19 crisis

Surprisingly, for Nigerian SMEs, the existential fears occasioned by COVID-19 relegated the adverse impacts (e.g., facilitation of fake news and misinformation) of EDCTs to the background. Misinformation through EDCT was less relevant during the COVID-19-induced disruption of SMEs SC, as SMEs used EDCTs to: (1) Become digital natives and digital adapters; and, (2) Acquire Digital Culturisation.

#### Become digital natives and digital adapters

Respondents affirm that the country’s weak institutional environment meant that dealing with misinformation was already the norm. Hence, developing resilience was more paramount than fake news. To do this, SMEs had to adjust to the digital landscape. As R26 and R1 comment:

*Now there must be a business rejig. In our business outlook, we must consider which clients will survive in the virtual world. We should know which clients we must retain. So, we start mapping across key indices that COVID-19 allows* (R26).

*We are trying to develop digital products and relying more on social media, e.g., Facebook or Instagram, in our publicity to allow us to remain in business and remain relevant* (R1).

#### Digital culturisation

Social media and virtual technologies have become more of a business culture for SMEs. The research participants posit that EDCTs allowed their business to stabilise even in the presence of fake news. R4 and R33 state thus:

*During COVID-19, we were less worried about fake news from social media. We tried to fit into the new culture or new normal. Social media, Instagram and virtual technologies have become a culture for any serious business. This is the future* (R4).

*Of course, we had loads of fake news on Facebook, but this is normal in our environment, where we cannot even trust the government. But, during COVID-19, I think business exigencies outweighed the pressures arising from negative or fake news. In Nigeria, the fear of our business failing overshadowed fake news* (R33).

Respondents confirm that *adapting to business exigencies was more important than misinformation* (R34). They acknowledge that fake news is associated with hedonic sensationalism, adding that survival was more paramount during COVID-19. As such, more and more SMEs are migrating to the digital space. Not only are SMEs now digital natives using various EDCTs, they are equally becoming digital adapters.

## Discussion

Our findings show that SMEs’ involvement in EDCT became prominent during COVID-19 SC disruptions. This is because problems arising from firms’ established cultural patterns and risk perceptions (DuHadway et al., 2018) meant that businesses required novel strategies to deal with SC disruptions prompted by COVID-19. We find that EDCTs were central in assisting SMEs manage challenges triggered by the pandemic (Borges et al., 2021). Our findings align with suggestions in Bagale et al., (2021) that, during COVID-19, the use of EDCTs became a strategic priority for SMEs. Our data show that despite the adversity induced by COVID-19, the diffusion of EDCTs brought unprecedented benefits in managing large and growing numbers of diverse relationships and information, qualities associated with resilience (Boin et al., 2010). In Nigeria, there is a perennial lack of access to finance, cumbersome sourcing of foreign exchange, and convoluted business processes. The diffusion of EDCTs enabled process diversification and facilitated resilience, allowing SMEs to reconfigure their resources to deal with emerging challenges (Aman & Seuring, 2021). Hence, in spite of the disruptions, SMEs could access new markets, finance and supplier credit.

Moreover, as Ivanov et al., (2014) and Kamalahmadi and Parast (2016) argue, our data show that SC flexibility is a crucial dimension in measuring organisational effectiveness and resilience. In Nigeria, the effects of COVID-19 and other institutional challenges (e.g., insecurity, bribery and corruption, poor transportation infrastructure, corruption, and other unethical business practices) (Transparency International, 2018) meant better organisation and resilience were essential. EDCTs enabled SMEs’ flexibility, allowing them to alter selling and buying platforms/outlets and redirect their businesses from B2B to B2C. Also, the EDCT-induced flexibility permitted efficient operations and cost savings in dealing with the SC disruptions triggered by COVID-19.

Resilience involves the ability to sustain reliable functioning in spite of challenging situations (Williams et al., 2017). SC collaboration originates in a paradigm of collaborative advantage (Scholten et al., 2014; Soni et al., 2014). From our findings, SMEs utilised EDCTs to develop SC collaborations. This form of open innovation (Iglesias et al., 2020; Markovic et al., 2021) unleashed benefits that include value add, increased learning and enhanced creativity as SC partners sought joint competitive advantages through collaboration (Scholten et al., 2014; Soni et al., 2014). During the pandemic, face-to-face connection with clients and work colleagues was not permitted, thereby disrupting lives and businesses. Considering their meagre resources, SMEs explored EDCTs to bond with their staff, clients, and suppliers. Thus, EDCT diffused, providing a platform for hope and faith, which helped build existing and new connections that deepen SMEs’ SC resilience.

**Fig. 2:**
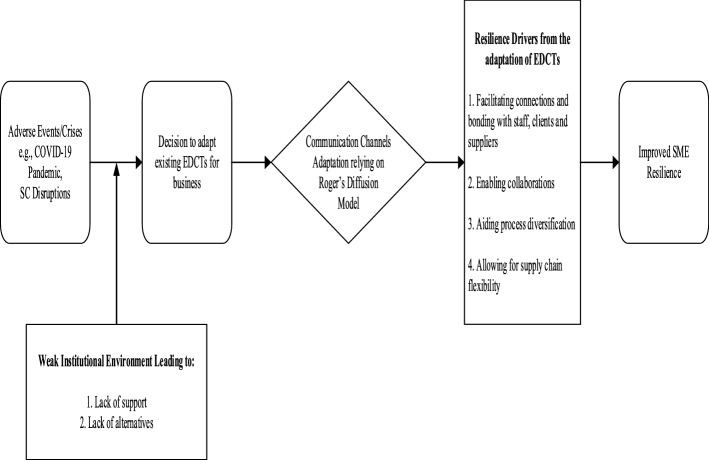
EDCT diffusion for SME resilience (created by the authors)

Overall, our findings show that the innovation needed to support resilience does not have to be costly (Masouras et al., 2021). However, given the uncertain future (Atwell et al., 2008) prompted by COVID-19, SMEs adapted the utility of existing EDCTs to build their resilience in the presence of weak institutions. This adaptation of EDCTs for SME resilience emerged from four resilience drivers—facilitating connections and bonding with staff, clients, and suppliers; enabling collaborations; aiding process diversification; and allowing SC flexibility (see Fig. 2 below). Figure 2 presents the EDCT diffusion for resilience, i.e., EDCTs facilitating SME resilience through relational adaptations in crisis times.

Therefore, rather than focus on the diffusion of innovation from a narrow perspective, this study builds on previous work (e.g., Vargo et al., 2015, 2020) that perceives diffusion of innovation as an institutionalisation process through which novel solutions emerge within a given context by deploying novel technologies such as EDCTs. This diffusion occurs as actors within a social system integrate and exchange existing resources to create additional value for themselves and others (Vargo et al., 2020). In addition, the literature (Pradhan et al., 2018; Rauch & Ansari, 2021) indicates that EDCTs influence social processes and affect human behaviours in societies. Thus, another important discovery from our data is that COVID-19 brought about a situation where SMEs changed their use of EDCTs from “for pleasure” to “for business”.

We can further infer from our data that existential fears led to the backgrounding of negative aspects of emerging technologies during the COVID-19 crisis. The emerging technologies innovation diffused into society, empowering SMEs to build resilience by becoming digital natives and adapters. In the same vein, our data reveals that institutional weaknesses in developing countries meant that SMEs could relegate the adverse impacts (e.g., fake news or miscommunication) of emerging technologies during COVID-19. Thus, while Kim and Dennis (2019) suggest that EDCTs are used more for hedonic purposes than business objectives, Nigerian SMEs adapted the technologies for business survival. Williams et al., (2017) identify cognitive, behavioural, emotion regulation, and relational resource endowments as the building blocks of durability capabilities. Likewise, as Soni et al., (2014) suggest, a risk management culture is an enabler for building resilience, and firms’ culture can be discussed concerning innovations (Kamalahmadi & Parast, 2016). In weak institutional environments (e.g., Nigeria), we opine that, because information asymmetry and distrust link with existing values and past experiences in society (Mochoge, 2014), the undesirable aspects of EDCTs, compared to their benefits, were deemed insignificant by SMEs.

## Contributions and conclusion

### Theoretical Contributions

This study addressed the question—*How did EDCTs enable SMEs in a weak institutional context to adapt their operations and build resilience during the COVID-19 pandemic-induced SC disruptions?* Our findings allow us to make three important theoretical contributions by linking emerging technologies and disruptions and the resilience of SMEs in a developing country. First, we applied the DOI by unpacking how the utility of EDCTs diffuses from the adoption stage as theorised in the Rogers (2003) five-stage innovation-decision model to adaptation and resilience. Specifically, we reveal how Nigerian SMEs adapted EDCTs to build resilience during COVID-19-induced SC disruptions. In doing this, our EDCT Diffusion–Resilience model aligns with the literature (e.g., Dearing & Cox, 2018; Vargo et al., 2015, 2020; David West et al., 2021) that theorises contextually about the utility of diffusion of innovation in an empirical way and with practical implications. Second, this research complements and links scholars’ efforts in the emerging digital technologies, crises, and resilience literature (e.g., Bagale et al., 2021; Herhausena et al., 2020; Kamalahmadi & Parast, 2016; Olan et al., 2022; Sebastian et al., 2017; Vial, 2019; Williams et al., 2017). In summary, we find that EDCTs enabled SMEs to be resilient amidst the SC disruptions through four drivers—facilitating connections and bonding with staff, clients, and suppliers; enabling collaborations; allowing process diversification; and, permitting SC flexibility.

This research reveals that during the COVID-19 pandemic, SMEs were surprisingly more concerned about building resilience than the impact of fake news on their operations. This suggests that SMEs are not overly concerned about EDCTs’ role in facilitating fake news and misinformation during a crisis. We show empirically that despite possible negative aspects of EDCTs, Nigerian SMEs would engage EDCTs to digitalise their businesses beyond using such technologies for hedonic purposes. This adaptation was induced by existential fears occasioned by limited government support. Focusing on weak institutional contexts helps advance the literature on deploying emerging technologies and innovations in developing countries. Scholars (e.g., Luo et al., 2021; Sengupta et al., 2021) have advocated the need to deepen understanding of institutional contexts to extend existing innovation themes and reveal alternative explanations for deploying EDCTs. In developing markets like Nigeria, where market regulations and transaction rules are inefficient (Luo et al., 2021), we advance the theorisation of adaptation of emerging technologies for SME resilience. Consistent with Pananod et al., (2020), this research suggests that EDCTs have lowered SC barriers for SMEs in developing economies. Hence, this research unpacks how the diffusion of emerging technologies can be adapted to enhance SMEs’ transactional competitiveness as well as deal with institutional disadvantages.

### Contributions to practice

First, the findings evidence an adaptive SC strategy that ensures better SMEs’ preparedness in crisis times. The EDCT–SME resilience drivers uncovered in this study offer operational guidance on how SMEs can adapt to SC disruptions. Considering the resource limitation challenges, our findings can be critical to SMEs’ survival, especially in weak markets. Therefore, we suggest that SME owners can enhance their resilience strategy by engaging the EDCT drivers unearthed in this research. For example, SMEs in weak institutional environments can be more resilient to SC disruptions through the driver of collaborations and partnerships. Due to the lack of institutional support, SMEs’ resilience strategy develops through collaborations to increase their organisational learning, creativity, and value add, which are critical for survival during SC disruptions.

Second, like Sengupta et al., (2021) report, this study highlights that EDCTs benefit economically disadvantaged SMEs as it helps minimise information asymmetry in the SC. EDCTs can help SMEs develop and gain competitive advantages (Bagale et al., 2021). Thus, we recommend that SMEs in weak institutional markets be more deliberate in deploying EDCTs for business operations.

### Limitations and future research

This paper has some limitations, which could be further explored and examined in future research. First, we interviewed only SME operators in a single country. This creates opportunities for a multi-country study on diffusing emerging technologies and their adaptation to SC disruptions in developing economies. Second, a comparative investigation of the utility of EDCTs in developed and developing markets could be another possible research direction, as their social and institutional variations could generate inconsistent outcomes as well as engender a greater understanding of EDCT utility.

## Conclusion

In conclusion, the COVID-19 crisis presented a situation where business activities could not continue as normal. Businesses had to adapt and build resilience in their SC to overcome these disruptions. This research reinforced the importance and relevance of EDCTs in dealing with SC disruptions (Kim & Dennis, 2019; Olan et al., 2022). Consistent with the literature (e.g., Endsley, 2018; Jayawickrama et al., 2019; Olan et al., 2022), this study demonstrates how EDCTs support SMEs in a developing country, especially in managing the dissemination of information during crises and enhancing their resilience potential.

In many developing countries, particularly in Africa, linking EDCTs to business performance is typically overlooked (Fougère & Meriläinen, 2021) as these technologies are used primarily for non-business purposes. Instructively, this study demonstrates that, despite SMEs’ limited resources, EDCTs assist SMEs in building their resilience. Therefore, in agreement with the nascent SC–resilience literature (e.g., Aman & Seuring, 2021; Bagale et al., 2021; Olan et al., 2022), we provide evidence of how EDCTs impact SMEs’ operations. More specifically, we employ Roger’s diffusion decision-making model to illustrate how changing EDCTs’ use from social to business helps SMEs cope with SC disruptions. This research strengthens the view that relational adaptation via EDCTs is fundamental to SMEs’ resilience during crises.

## Appendix 1: Interview questions

1. How has COVID-19 impacted your business, suppliers (local and foreign), and your relationships with your business partners generally?
2. Was there any disruption to your business? Disruption can be a disruption of information. How did COVID-19 affect your relationship with the businesses you deal with?
3. How did you get the business running during the crisis? What exactly did you do as a business to make sure you kept making revenue?
4. Do you utilise communication technologies (e.g., social media and virtual technologies) in your business? Did you use these communications technologies during COVID-19? How did these communications technologies affect your business plans, positively or negatively?
5. When the COVID-19 is over, what do you plan to do about your business? How will you make sure you stay in business? How will you use all these new communications technologies?
6. What were the critical factors that helped your business cope with the COVID-19 crisis (and any other crisis your business has faced)?
7. How influential was government or government institutions during the crisis for your business?
8. How prepared is your business prepared for any future crisis?
9. Is there anything else you would like to tell me about disruptions to your business relationships, supply chains, COVID-19, or Government interventions that I might not have covered?

## Footnotes

1 Supply chain (SC) disruptions present themselves in various forms (e.g., poor communication, transportation delays, quality issues, supply shortage, IT failures) (Williams & You, 2018; Blackhurst et al., 2005).

2 Emerging digital technologies are new technologies (e.g., social media, artificial intelligence, virtual technologies, blockchain, etc.) which facilitate information and communication and are useful for firms’ administrative changes (Sebastian et al., 2017; Vial, 2019).

3 Weak institutional countries are those with poor regulatory quality, exhibit high corruption levels, and present low government effectiveness (Kaufmann et al., 2008).

4 The term ‘fake news’ is used interchangeably to describe news designed for gaining financial profit or discrediting others (Allcott & Gentzkow, 2017), distorted facts (Tandoc et al., 2018), or uncomfortable or disagreeable news (Lilleker, 2017).

5 The EU and UK define an SME as a business with less than 250 employees and less than 50 M Euro turnover.

6 Compliance included submission of annual financial reports. We obtained recent financial statements from all SMEs except two who informed us they needed board approval before sharing these with us.

7 Our sample selection was based on the need to have a diffusion of innovation theorisation and not statistical sampling as we were not in pursuit of generalizability of the findings (Farquhar et al., 2020) nor was it sector-specific.

8 Most SMEs utilised contract/ad hoc staff regularly.

9 Even though as at the time of data collection, most restrictions had been lifted with activities back to pre-COVID-19 levels, social distancing and other health concerns meant face-to-face interviews were not advisable. 40 interviews were conducted via Zoom with cameras on, hence some of the advantages of face-to-face interviews were still applicable.

10 A considerable volume of literature in qualitative research suggests that ‘how many’ is not what matters (Mason, 2010). A researcher should, therefore, aim to satisfy himself/herself that he/she has learned, and understands the phenomenon, enough to enable knowledge generation. This was the basis for determining the appropriate sample size.

## References

[CR1] Adegbite E (2015). Good corporate governance in Nigeria: Antecedents, propositions and peculiarities. International Business Review.

[CR2] Allcott H, Gentzkow M (2017). Social media and fake news in the 2016 election. Journal of Economic Perspectives.

[CR3] Aman S, Seuring S (2021). Analysing developing countries approaches of supply chain resilience to COVID-19. The International Journal of Logistics Management.

[CR4] Ambulkar S, Blackhurst J, Grawe S (2015). Firm's resilience to supply chain disruptions: Scale development and empirical examination. Journal of Operations Management.

[CR5] Ashiru F, Adegbite E, Nakpodia F, Koporcic N (2022). Relational governance mechanisms as enablers of dynamic capabilities in Nigerian SMEs during the COVID-19 crisis. Industrial Marketing Management.

[CR6] Atwell RC, Schulte LA, Westphal LM (2008). Linking resilience theory and diffusion of innovations theory to understand the potential for perennials in the U.S. Corn Belt. Ecology and Society.

[CR7] Baccarella CV, Wagner TF, Kietzmann JH, McCarthy LP (2018). Social media? It's serious! Understanding the dark side of social media. European Management Journal.

[CR8] Bagale G, Vandadi V, Singh D, Sharma D, Garlapati D, Bommisetti R, Gupta R, Setsiawan R, Subramaniyaswamy V, Sengan S (2021). Small and medium-sized enterprises' contribution in digital technology. Annals of Operations Research.

[CR9] Bak O, Shaw S, Colicchia C, Kumar V (2020). A Systematic literature review of supply chain resilience in small-medium enterprises (SMEs): A call for further research. EEE Transactions on Engineering Management.

[CR10] Baum G, Sendler U (2013). Innovation also basis der nächsten industrie revolution. Industries 4.0.

[CR11] Berthon PR, Pitt LF (2018). Brands, truthiness and post-fact: Managing brands in a post-rational. World Journal of Macromarketing.

[CR12] Berthon PR, Pitt LF, McCarthy I, Kates S (2007). When customers get clever: Managerial approaches to dealing with creative consumers. Business Horizons.

[CR13] Binham C (2019). Companies fear rise of fake news and social media rumours.

[CR14] Blackhurst J, Craighead CW, Elkins D, Handfield RB (2005). An empirically derived agenda of critical research issues for managing supply-chain disruptions. International Journal of Production Research.

[CR201] Boin, A., Comfort, L. K., & Demchak, C. C. (2010). The Rise of Resilience. In L. K. Comfort, A. Boin, & C. C. Demchak (Eds.), *Designing resilience: Preparing for extreme events* (pp. 1–12). Pittsburgh, USA: University of Pittsburgh Press.

[CR15] Borges AFS, Laurindo FJB, Spínola MM, Gonçalves RF, Mattos CA (2021). The strategic use of artificial intelligence in the digital era: Systematic literature review and future research directions. International Journal of Information Management.

[CR16] Bradley E, Curry L, Devers K (2007). Qualitative Data Analysis for Health Services Research: Developing Taxonomy, Themes, and Theory. Health Services Research.

[CR200] Brandon-Jones, E., Squire, B., Autry, C. W., & Petersen, K. J. (2014). A contingent resource-based perspective of supply chain resilience and robustness. *Journal of Supply Chain Management, 50*(3), 55–73.

[CR17] Burnard P (1991). A method of analysing interview transcripts in qualitative research. Nurse Education Today.

[CR18] Campbell J, Quincy C, Osserman J, Pedersen O (2013). Coding in-depth semi structured interviews: Problems of unitisation and intercoder reliability and agreement. Sociological Methods & Research.

[CR19] Cenamor J, Parida V, Wincent J (2019). How entrepreneurial SMEs compete through digital platforms: The roles of digital platform capability, network capability and ambidexterity. Journal of Business Research.

[CR20] Chesbrough H (2020). To recover faster from Covid-19, open up: Managerial implications from an open innovation perspective. Industrial Marketing Management.

[CR21] David-West O, Oni O, Ashiru F (2021). Diffusion of innovations: Mobile money utility and financial inclusion in Nigeria. Insights from agents and unbanked poor end users. Information Systems Frontiers.

[CR22] Day JM, Melnyk SA, Larson PD, Davis EW, Whybark DC (2012). Humanitarian and disaster relief supply chains: A matter of life and death. Journal of Supply Chain Management.

[CR23] Dearing JW, Cox JG (2018). Diffusion Of innovations theory, principles. And Practice. Health Affairs.

[CR24] Dey I (2003). Qualitative data analysis: A user friendly guide for social scientists.

[CR25] Di Domenico G, Sit J, Ishizaka A, Nunan D (2021). Fake news, social media and marketing: A systematic review. Journal of Business Research.

[CR26] DuHadway S, Carnovale S, Kannan V (2018). Organisational communication and individual behavior: Implications for supply chain risk management. Journal of Supply Chain Management.

[CR27] Eggers F (2020). Masters of disasters? Challenges and opportunities for SMEs in times of crisis. Journal of Business Research.

[CR28] Eisenhardt KM (1989). Building up theories from case study research. The Academy of Management Review.

[CR29] Endsley MR (2018). Combating information attacks in the age of the internet: New challenges for cognitive engineering human factors. THe Journal of Human Factors and Ergonomics Society.

[CR30] Farquhar J, Michels N, Robson J (2020). Triangulation in qualitative case study research: Widening the scope. Industrial Marketing Management.

[CR31] Fischer E, Reuber AR (2011). Social Interaction via new social media: (How) Can interactions on twitter affect effectual thinking and behavior?. Journal of Business Venturing.

[CR32] Flick U (2014). An introduction to qualitative research.

[CR33] Fosso Wamba S, Carter L (2017). Social media tools adoption and use by SMEs: An empirical study. Journal of End User and Organizational Computing.

[CR34] Fougère M, Meriläinen E (2021). Exposing three dark sides of social innovation through critical perspectives on resilience. Industry and Innovation.

[CR35] Franceschinis C, Thiene M, Scarpa R, Rose J, Moretto M, Cavalli R (2017). Adoption of renewable heating systems: An empirical test of the diffusion of innovation theory. Energy.

[CR36] Gabler C, Richey R, Stewart G (2017). Disaster Resilience through public-private short-term collaboration. Journal of Business Logistics.

[CR37] Gioia D, Corley K, Hamilton A (2012). Seeking qualitative rigor in inductive research: Notes on the Gioia methodology. Organizational Research Methods.

[CR38] Gioia D, Price K, Hamilton A, Thomas J (2010). Forging an identity: An insider-outsider study of processes involved in the formation of organisational identity. Administrative Science Quarterly.

[CR39] Gunasekaran A, Rai B, Griffin M (2011). Resilience and competitiveness of small and medium size enterprises: An empirical research. International Journal of Production Research.

[CR40] Hazen BT, Boone CA, Ezell JD, Jones-Farmer LA (2014). Data quality for data science, predictive analytics, and big data in supply chain management: An introduction to the problem and suggestions for research and applications. International Journal of Production Economics.

[CR41] Hendricks K, Singhal V (2005). An Empirical analysis of the effect of supply chain disruptions on long-run stock price performance and equity risk of the firm. Production and Operations Management.

[CR42] Herhausena D, Miocevic D, Morgan RE, Kleijnen MHP (2020). The digital marketing capabilities gap. Industrial Marketing Management.

[CR43] Hohenstein N, Feise E, Hartmann E, Giunipero L (2015). Research on the phenomenon of supply chain resilience: A systematic review and paths for further investigation. Int. J. Phys. Distrib. Logistics Manage..

[CR44] Hsieh H, Shannon S (2005). Three approaches to qualitative content analysis. Qualitative Health Research.

[CR45] Huber G, Power D (1985). Retrospective reports of strategic level managers. Strategic Management Journal.

[CR46] Iglesias O, Markovic S, Bagherzadeh M, Singh J (2020). Co-creation: A key link between corporate social responsibility, customer trust, and customer loyalty. Journal of Business Ethics.

[CR47] Ivanov D, Sokolov B, Dolgui A (2014). The ripple effect in supply chains: Trade-off 'efficiency-flexibility-resilience' in disruption management. International Journal of Production Research.

[CR48] Jayawickrama U, Liu S, Hudson Smith M, Akhtar P, Al Bashir M (2019). Knowledge retention in ERP implementations: The context of UK SMEs. Production Planning & Control.

[CR49] Johnson TJ, Kaye BK (2015). Reasons to Believe: Influence of credibility on motivations for using social networks. Computers in Human Behavior.

[CR50] Kamalahmadi M, Parast M (2016). A review of the literature on the principles of enterprise and supply chain resilience: Major findings and directions for future research. Int. J. Production Economics.

[CR51] Kaplan AM, Haenlein M (2010). Users of the world, Unite! The challenges and opportunities of social media. Business Horizons.

[CR52] Kaufmann, D., Kraay, A., & Mastruzzi, M. (2008). Governance matters VII: Aggregate and individual governance indicators for 1996–2007*.**World Bank Policy Research Working Paper No. 4654*. Retrieved on August 10, 2018, from http://papers.ssrn.com/sol3/Papers.cfm?abstract_id=1148386.

[CR53] Khanna T, Palepu K, Sinha J (2005). Strategies that fit emerging markets. Harvard Business Review.

[CR54] Kim A, Dennis AR (2019). Says who? The effects of presentation format and source rating on fake news in social media. MIS Quarterly: Management Information Systems.

[CR55] Koporcic N, Tolusic Z, Tolusic Z (2015). Introducing the interaction approach for successful business relationships. Ekonomski Vjesnik/Econviews-Review of Contemporary Business, Entrepreneurship and Economic Issues.

[CR56] Lilleker, D. (2017). Evidence to the culture, media and sport committee's* Fake news' inquiry, Bournemouth University*, retrieved from http://eprints.bournemouth.ac.uk/28610/3/Evidence%20Submission%20-%20Fake%20News%20FINAL.pdf (November 14, 2021).

[CR57] Lloyds, P. (2018). *Emerging economies have $160bn insurance gap*. Emerging economies have $160bn insurance gap—Lloyds (lloyds.com). Accessed July 30, 2022.

[CR58] Lobe B, Morgan D, Hoffman K (2020). Qualitative data collection in an era of social distancing. International Journal of Qualitative Methods.

[CR59] Luo X, Cao D, Tjahjono B, Adegbile A (2021). Business model innovation themes of emerging market enterprises: Evidence in China. Journal of Business Research.

[CR60] Markovic S, Koporcic N, Arslanagic-Kalajdzic M, Kadic-Maglajlic S, Bagherzadeh M, Islam N (2021). Business-to-business open innovation: COVID-19 lessons for small and medium-sized enterprises from emerging markets. Technological Forecasting and Social Change.

[CR61] Mason M (2010). Sample size and saturation in PhD studies using qualitative interviews. Forum Qualitative Social Research.

[CR62] Masouras A, Pistikou V, Komodromos M, Apostolopoulos N, Chalvatzis K, Liargovas P (2021). Innovation analysis in cypriot small and medium-sized enterprises and the role of the European Union. Entrepreneurship, Institutional Framework and Support Mechanisms in the EU.

[CR63] Mochoge OC (2014). SMES' Adoption of Web-based Marketing: Empirical Evidence from Kenya. International Journal of Computer Science. Issues (Chicago, Ill.).

[CR64] Modrak, V., Soltysova, Z., & Poklemba, R. (2019) Mapping requirements and roadmap definition for introducing I 4.0 in SME Environment. In *Advances in Manufacturing Engineering and Materials,* (pp. 183–194). Cham: Springer

[CR65] Nakpodia F, Shrives P, Sorour K (2020). Examining the link between religion and corporate governance practice: Insights from Nigeria. Business and Society.

[CR66] Daily Trust Newspapers (2021). *Nine Things You Need to Know About E-Naira*. https://dailytrust.com/nine-things-you-need-to-know-about-e-naira. Accessed November 24, 2021.

[CR67] Nigerian Bureau of Statistics (2021). national survey of micro small & medium enterprises (MSMEs) 2017. https://nigerianstat.gov.ng/elibrary?queries[search]=enterprises. (February 25, 2021).

[CR68] Olan F, Arakpogun E, Jayawickrama U, Suklan J, Liu S (2022). Sustainable supply chain finance and supply networks: The role of artificial intelligence. IEEE Transactions on Engineering Management.

[CR69] Omotosho B (2020). Small scale craft workers and the use of social media platforms for business performance in southwest Nigeria. Journal of Small Business & Entrepreneurship.

[CR70] Oni O, Camilleri MA (2021). Small- and medium-sized enterprises' engagement with social media for corporate communication. Strategic corporate communication in the digital age.

[CR71] Oyemomi O, Liu S, Neaga I, Alkhuraiji A (2016). How knowledge sharing and business process contribute to organisational performance: Using the fsQCA approach. Journal of Business Research.

[CR72] Pananod P, Gereffi G, Pedersen T (2020). An integrative typology of global strategy and global value chains: The management and organisation of cross-border activities. Global Strategy Journal.

[CR73] Papadopoulos T, Gunasekaran A, Dubey R, Altay N, Childe S, Fosso-Wamba S (2017). The role of Big Data in explaining disaster resilience for sustainability. Journal of Cleaner Production.

[CR74] Pettit T, Croxton K, Fiksel J (2013). Ensuring supply chain resilience: Development and implementation of an assessment tool. Journal of Business Logistics.

[CR75] Pettit T, Croxton K, Fiksel J (2019). The evolution of resilience in supply chain management: A retrospective on ensuring supply chain resilience. Journal of Business Logistics.

[CR76] Polanco-Diges L, Debasa F (2020). The use of digital marketing strategies in the sharing economy: A literature review. Journal of Spatial and Organizational Dynamics.

[CR77] Polat V, Yarimoglu E (2018). Why and How Small and medium-sized enterprises use social media. Journal of Business in the Digital Age.

[CR78] Polit D, Beck C (2012). Nursing research: Principles and methods.

[CR79] Pradhan P (2018). Digital marketing and SMEs: An Identification of research gap via Archives of past research. Journal of Internet Banking and Commerce.

[CR80] Prescott M, Conger S (1995). Information technology innovations: A classification by IT locus of impact and research approach. Database for Advances in Information Systems.

[CR81] Rajesh R (2021). Optimal trade-offs in decision-making for sustainability and resilience in manufacturing supply chains. Journal of Cleaner Production.

[CR82] Rauch M, Ansari S (2021). Waging war from remote cubicles: How workers cope with technologies that disrupt the meaning and morality of their work. Organization Science.

[CR83] Rehman A, Jajja MSS, Khalid RU, Seuring S (2020). The impact of institutional voids on risk and performance in base-of-the-pyramid supply chains. The International Journal of Logistics Management.

[CR84] Rice JB, Caniato F (2003). Building a secure and resilient supply network. Supply Chain Manag. Rev..

[CR85] Rogers, E. M. (1983). *Diffusion of innovation* (3rd ed). Free Press. Science, 359(6380), pp. 1094–1096.

[CR86] Rogers EM (1995). Diffusion of innovations.

[CR87] Rogers EM (2003). Diffusion of Innovation.

[CR88] Scholten K, Schilder S (2015). The role of collaboration in supply chain resilience. Supply Chain Management: An International Journal.

[CR89] Scholten K, Scott P, Fynes B (2014). Mitigation processes—antecedents for building supply chain resilience. Supply Chain Management: An International Journal.

[CR90] Sebastian I, Ross J, Beath C, Mocker M, Moloney K, Fonstad N (2017). How big old companies navigate digital transformation. MIS Quarterly.

[CR91] Sengupta T, Narayanamurthy G, Moser R, Pereira V, Bhattacharjee D (2021). Disruptive technologies for achieving supply chain resilience in COVID-19 Era: An implementation case study of satellite imagery and blockchain technologies in fish supply chain. Information Systems Frontiers.

[CR92] Soni S, Jain V, Kumar S (2014). Measuring supply chain resilience using a deterministic modeling approach. Computers & Industrial Engineering.

[CR93] Spath D, Ganschar O, Gerlach S, Hämmerle TK, Schlund S (2013). Produktionsarbeit der Zukunft – Industrie 40.

[CR94] Srinivasan R, Bajaj R, Bhanot S (2016). Impact of social media marketing strategies used by micro small and medium enterprises (MSMEs) on Customer acquisition and retention. IOSR Journal of Business and Management.

[CR95] Tajvidi M, Richard MO, Wang Y, Hajli N (2018). Brand co-creation through social commerce information sharing: The role of social media. Journal of Business Research.

[CR96] Talebian A, Mishra S (2018). Predicting the adoption of connected autonomous vehicles: A new approach based on the theory of diffusion of innovations. Transportation Research Part C: Emerging Technologies.

[CR97] Tandoc E, Ling R, Westlund O, Duffy A, Goh D, Zheng W (2018). Audiences' acts of authentication in the age of fake news: A conceptual framework. New Media & Society.

[CR98] Transparency International (2018). *Corruption Index.* Retrieved July 17, 2019, from https://www.transparency.org/files/content/pages/2018_CPI_Executive_Summary.pdf.

[CR99] Tukamuhabwa B, Stevenson M, Busby J (2017). Supply chain resilience in a developing country context: A case study on the interconnectedness of threats, strategies and outcomes. Supply Chain Management.

[CR100] Van Slyke C, Belanger F, Comunale C (2004). Factors influencing the adoption of Web-based shopping: The impact of trust. Database for Advances in Information Systems.

[CR101] Van Slyke C, Lou H, Day J (2002). The impact of perceived innovation characteristics on intention to use groupware. Information Resource Management Journal.

[CR102] Vargo S, Akaka M, Wieland H (2020). Rethinking the process of diffusion in innovation: A service-ecosystems and institutional perspective. Journal of Business Research.

[CR103] Vargo S, Wieland H, Akaka M (2015). Innovation through institutionalisation: A service ecosystems perspective. Industrial Marketing Management.

[CR104] Vial G (2019). Understanding digital transformation: A review and a research agenda. The Journal of Strategic Information Systems.

[CR105] Waller M, Fawcett S (2013). Data science, predictive analytics, and big data: A revolution that will transform supply chain design and management. Journal of Business Logistics.

[CR106] Weber R (1990). Basic content analysis.

[CR107] Williams C, You JJ (2018). Building resilience in client organisations: The consultant's challenge. Management Consulting Journal.

[CR108] Williams T, Sutcliffe K, Shepherd D, Zhao E (2017). Organisational response to adversity: Fusing crisis management and resilience research streams. Academy of Management Annals.

[CR109] World Bank (2020). The world bank indicator. Retrieved December 23, 2020, from https://data.worldbank.org/indicator/NY.GDP.MKTP.CD?locations=NG.

[CR110] You, J. J. (2021). *Exploring Organisational Resilience from an Inter-organisational Perspective: Relational Resilience based on Business Ecosystems in China*. Doctoral dissertation, Durham University

